# Social support as a mediator of the relationship between forgiveness and post-traumatic growth in hemodialysis patients: A structural equation modeling approach

**DOI:** 10.3389/fpsyt.2022.974045

**Published:** 2022-12-08

**Authors:** Yansheng Ye, Zongwu Tong, Changqiong Li, Xiufang Gao, Yane Sun, Jianqing Xu, Qian Xu, Chengrong Song

**Affiliations:** Sixth Affiliated Hospital of Kunming Medical University, The People’s Hospital of Yuxi City, Yuxi, Yunnan, China

**Keywords:** chronic kidney disease, forgiveness, social support, post-traumatic growth, hemodialysis patients, structural equation modeling

## Abstract

**Background:**

Post-traumatic growth (PTG) refers to the positive psychological changes experienced with individuals after struggling with highly challenging life circumstances. Forgiveness can facilitate positive outcomes such as reduced distress, anxiety, and depression. Many studies have tested the relationships among forgiveness, social support, and PTG; however, a mechanism of social support has not been completely explored in hemodialysis patients.

**Objective:**

To test the relationship between forgiveness and post-traumatic growth and verify the mediating factor of social support on the relationship between forgiveness and PTG in hemodialysis patients.

**Materials and methods:**

In a descriptive cross-sectional study using convenience sampling from March to May 2021, 497 hemodialysis patients from nine hospitals filled out the Perceived Social Support Scale (PSSS), Heartland Forgiveness Scale (HFS), Post-traumatic Growth Inventory (PTGI), and general information. Data were analyzed using SPSS, and structural equation modeling was used to explore the relationships among forgiveness, social support, and PTG.

**Results:**

Forgiveness was significantly positively associated with PTG (*P* < 0.01). The proposed model provided a good fit to the data. Social support was found to play a partial mediating role between forgiveness and PTG (a*b = 0.122, BCa 95% CI: 0.078∼0.181).

**Conclusion:**

The results imply that forgiveness significantly directly and indirectly is related to PTG. Forgiveness in hemodialysis patients should be detected and effectively managed to ameliorate positive effects on PTG. It is necessary for nurses to consider implementing forgiveness interventions with an emphasis on building social support strategies to help hemodialysis patients enhance their PTG.

## Introduction

Chronic kidney disease (CKD), classified into five stages, is a public health problem worldwide that seriously damages human health. The prevalence of CKD has been reported as 13.4% in the world and 13.2% in Chinese adults (1, 2). End-stage renal disease (ESRD), the final stage of CKD, is characterized by irreversible kidney function damage, and its prevalence is increasing year-by-year (3). Maintenance hemodialysis (MHD) is a primary and commonly used therapeutic approach for ESRD patients in China (4). Hemodialysis is performed by over 713,000 people according to the Chinese Renal Data System (5).

End-stage renal disease patients with MHD are often perceived as having experienced a potentially traumatic event and suffer from both physical and mental problems, such as anxiety and depression (6). Post-traumatic growth (PTG) refers to the positive psychological changes experienced with individuals after struggling with highly challenging life circumstances (7). The advantages of PTG to the rehabilitation and psychological adaptation of patients experiencing a serious traumatic event include fewer depressive symptoms, enhanced psychological wellbeing, better adherence to treatment, better perceived physical health, and better health-related quality of life (8–10).

Most studies of the relationship between forgiveness and PTG have found a positive relationship. For example, Su et al. (11) found that forgiveness was positively correlated with PTG (*P* < 0.01) and that forgiveness significantly predicted PTG (*P* < 0.05) among burn survivors. Martinčeková et al. (12) reported that forgiveness was positively associated with PTG (*P <* 0.01) in a sample of grieving mothers. Chang et al. (13) found that people experiencing Aceh conflict with forgiveness were significantly positively correlated with PTG (*P <* 0.01). One study found that the relationship between forgiveness and PTG was not statistically significant (*P* > 0.05) (14). These inconsistent results regarding the relationship between forgiveness and PTG are worth deserving empirical clarification in order to develop more effective methods for intervention.

A growing body of research demonstrates that perceived social support from others can be positively associated with forgiveness. Social support refers to perceived assistance from others, including tangible, emotional, and informational assistance (15). Weinberg (16), through an investigation of 108 injured terrorism attack survivors aged between 21 and 70 years old, found social support to be positively correlated with forgiveness (*P* < 0.01), meaning that the stronger social support was, the greater the level of forgiveness was. Similarly, Tian et al. (17) investigated 713 older people and found social support to be positively correlated with forgiveness (*P <* 0.01). The literature shows that higher levels of perceived social support play a positive and protective role in better adaptation to society when experiencing negative events (18). Therefore, people’s perceived social support may have a positive correlation with forgiveness in general practice. However, few studies have verified the relationship between social support and forgiveness in hemodialysis patients.

According to qualitative and quantitative studies, PTG can be improved through social support, which is viewed as coping assistance (19–21). Social support is an important factor in alleviating distress and improving one’s capacity to adapt to traumatic events (22). For instance, higher levels of social support was found to potentially predict fewer post-traumatic stress reactions in a sample of adolescent victims of sexual abuse (23). Panjikidze et al. (24) reported that a main predictor of PTG is social support and that informational support from peers is significantly positively related to PTG (*P* < 0.01). In a meta-analytical study by Prati et al. (25) on factors contributing to PTG, social support (received support, perceived support, and satisfaction with social support) was found to predict PTG. Social support, a supportive social resource, may reduce levels of anxiety, depression, and other mental and physical disorders in samples of those exposed to a range of traumatic events (26, 27).

**FIGURE 1:**
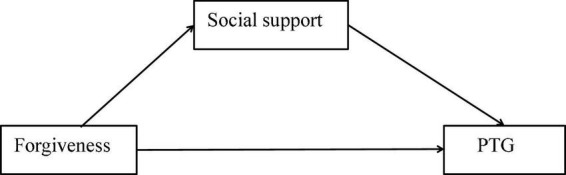
Hypothesized theoretical model.

A growing body of studies has tested the relationships between forgiveness, social support, and PTG; however, the mechanism of social support has not been fully explored. Social support as a mediator variable was based on the stress-and-coping theory of forgiveness and empirical evidence acquired from the aforementioned studies (24, 28, 29). For instance, Xu et al. (29) investigated 443 middle school students and found social support as a mediator between deliberate rumination and PTG. Similarly, Panjikidze et al. (24) found that social support significantly mediated the relationship between personality factors and PTG. The hypothesized model developed in this study is as follows (Figure 1). The model was used to explore the relationships between forgiveness, social support, and PTG, with the results suggesting that forgiveness influences PTG through its effects on social support. However, there are few studies on how social support enhances the relationship between forgiveness and PTG, and it remains an undervalued and understudied topic.

On the above conceptual bases, the purpose of this study is (1) to examine the relationship between forgiveness and PTG in hemodialysis patients and (2) to confirm the mediating effect of social support on the relationship between forgiveness and PTG.

## Materials and methods

### Study design and setting

A cross-sectional design was used. The participants were recruited from nine hospitals (one third-level general hospitals and eight second-level general hospitals) in the city of Yuxi in Yunnan Province in China from March to May 2021. The questionnaire including demographic information, disease-related information, forgiveness, social support, and PTG, which lasted 25∼30 min, was distributed to the hemodialysis patients.

### Participants

Participants meeting the following inclusion criteria were accepted for this study: (1) CKD5 stage status, (2) hemodialysis performed 2∼3 times a week, (3) hemodialysis lasting over 3 months, (4) informed consent to participate in this study, and (5) age of 18 years or more. The exclusion criteria were as follows: (1) having an infectious disease, including syphilis, HIV, and hepatitis B, and (2) the inability to communicate or read and write normally.

Sample sizes of 200 or more should generally be obtained for SEM or 5∼10 times the number of parameters should be estimated (30). With 11 observed variables, the number of parameters estimated for this study was 54; thus, the inclusion of 497 hemodialysis patients was in line with the above rules.

### Ethics statement

The Committee of Medical Ethics of the Sixth Affiliated Hospital of Kunming Medical University approved this research (Ethics number: 2020 kmykdx6937). After providing the hemodialysis patients with a sufficient explanation of the study, written informed consent was obtained. The participants were ensured of the confidentiality of the collected information.

### Measures

#### Demographic characteristics and disease-related information

A personal information questionnaire was used to collect the participants’ demographic information, including information on gender, age, marital status, income status, religious belief, and education. Disease-related information regarding primary kidney disease and the number of complications was obtained from the participants’ medical records. Gender was divided into two categories, i.e., “male” and “female.” Number of complications was grouped into “0,” “1,” and “≥2.” The categories of the rest of indicators are depicted in Table 1.

**TABLE 1:** Demographic characteristics and disease-related information and distributions of post-traumatic growth (PTG) among hemodialysis patients (*N* = 497).

Variable	*N* (%)	PTG (Mean ± SD)
**Gender**		
Male	302 (60.76)	3.80 ± 0.82
Female	195 (39.24)	3.97 ± 0.72**
**Age, years**		
18–45	163 (32.80)	3.81 ± 0.81
45–97	334 (67.20)	3.90 ± 0.78
**Education**		
<High school diploma	368 (74.04)	3.88 ± 0.77
≥High school diploma	129 (25.96)	3.85 ± 0.83
**Marital status**		
Married	402 (80.89)	3.82 ± 0.76
Others	95 (19.11)	3.77 ± 0.86
**Income status (per month)**		
<5,000 Yuan	396 (79.68)	3.83 ± 0.81
5,000–10,000 Yuan	86 (17.30)	4.02 ± 0.68
≥10,000 Yuan	15 (3.02)	4.20 ± 0.50*
**Religious belief**		
Yes	54 (10.87)	3.75 ± 0.78
No	443 (89.13)	3.88 ± 0.79
**Primary kidney disease**		
Hypertension	153 (30.79)	3.88 ± 0.80
Primary glomerular disease	151 (30.38)	3.81 ± 0.82
Diabetes mellitus	107 (21.53)	3.90 ± 0.82
Others	86 (17.30)	3.91 ± 0.73
**Number of complications**		
0	114 (22.94)	3.83 ± 0.84
1	89 (17.91)	4.09 ± 0.74*
≥2	294 (59.15)	3.82 ± 0.77

**P < 0.01, *P < 0.05.

#### Measurement of forgiveness

The Heartland Forgiveness Scale (HFS) was first developed by Thompson et al. (31) to measure the forgiveness levels of subjects. The Chinese version of the HFS developed by Zhang (32) includes 14 items and three subscales: forgiveness of self (four items), forgiveness of others (five items), and forgiveness of situations (five items). Each item is rated on a seven points scale ranging from 1 (complete non-conformity) to 7 (complete conformity). HFS scoring uses three subscales ranging from 14 to 98. As the total score increases, the level of forgiveness also increases. The HFS is a self-rated scale. The Chinese version of HFS has been validated as a instrument with good reliability and validity for measuring the forgiveness levels among Chinese hemodialysis patients (4). In this study, the Cronbach’s alpha coefficient for this instrument was measured as 0.869.

#### Measurement of social support

The Perceived Social Support Scale (PSSS) was first developed by Zimet et al. (33) to measure the perceived social support levels of subjects. The Chinese version developed by Jiang (34) includes 12 items and three subscales: family support (four items), friends support (four items), and other support (four items). Each item is rated on a seven points scale, ranging from 1 (strongly disagree) to 7 (strongly agree). PSSS scoring uses three subscales ranging between 12 and 84. As the total score increases, the level of perceived social support also increases. The PSSS is a self-rated scale. The Chinese version of PSSS with good validity and reliability has been widely used in China (17). In this study, the Cronbach’s alpha coefficient for this instrument was measured as 0.876.

#### Measurement of post-traumatic growth

The Post-traumatic Growth Inventory (PTGI) was first developed by Tedeschi et al. (35) to measure PTG levels in subjects. The Chinese version developed by Wang et al. (36) includes 20 items and five subscales: new possibilities (three items), appreciation of life (four items), spiritual change (three items), personal strength (four items), and relating to others (six items). Each item is rated on a six points scale ranging from 0 to 5. PTGI scoring uses five subscales of between 0 and 100. As the total score increases, the level of post-traumatic growth also increases. The PTGI is a self-rated scale. The Chinese version of PTGI showed to be valid and reliable in Chinese people suffered traumatic events (9). In this study, the Cronbach’s alpha coefficient for this instrument was measured as 0.886.

### Data analysis

#### Primary analysis

The data for forgiveness, social support, and PTG show normal distributions (tested by a Kolmogorov–Smirnov test with SPSS version 25.0). The differences in PTG among categorical groups were tested using *t*-tests and one-way ANOVA. The correlations among three variables of PTG, forgiveness and social support were explored using Pearson correlation analysis. Statistical significance was defined as *P* < 0.05 (two-tailed).

#### Hierarchical multiple regression analysis

Hierarchical multiple regression (HMR) analysis was used to explore the predictors of PTG and verify the mediating effect of social support on the relationship between forgiveness and PTG. The asymptotic and resampling methods developed by Preacher and Hayes (37) were used in this study to explore social support as a potential mediator on the association between forgiveness and PTG. PTG was used as a dependent variable and the independent variables (i.e., demographic information and disease-related information, forgiveness, and social support) were entered in three steps as follows: Step 1: demographic information and disease-related information of hemodialysis patients; Step 2: forgiveness; and Step 3: social support. If the regression coefficient of forgiveness to the PTG was significant and reduced from step 2 to step 3, there was a partial mediating effect. If the regression coefficient of forgiveness to the PTG was not statistically significant (*P* > 0.05), it indicated that social support has a complete mediating effect. The analysis was carried out in stages by successively inputting blocks of independent variables in the model.

#### Structural equation modeling analysis

Structural equation modeling (SEM) was used to confirm the mediating effect of social support on the relationship between forgiveness and PTG which was analyzed by Amos 25.0. The model fitted with the SEM criteria (SRMR < 0.08, RMSEA < 0.08, CFI > 0.90, TFI > 0.90, GFI > 0.90, and *x*^2^/d*f* < 5). Sober test was used to test whether the mediating effect of social support was statistically significant. Bootstrapping was used to explore the mediator (a*b product) of social support on the relationship between forgiveness and PTG, with the estimates of 1,000 samples and 95% bias corrected and accelerated confidence intervals. If the value of zero was outside confidence interval, the significance of a mediating effect was ascertained.

## Results

### Demographic characteristics and disease-related information

The demographic characteristics and disease-related information of the hemodialysis patients are presented in Table 1. The sample comprised 497 patients, and the respondents’ mean age was 50.89 years (SD = 13.90; range 19–97); 60.76% of the respondents were male (*n* = 302), 67.20% were 45–97 years old, 74.04% had less than a high school diploma, 80.89% were married, 79.68% had an income (per month) of less than 5000 Yuan, 89.13% had no religious affiliation, 30.79% with primary kidney disease had hypertension, and 59.15% had experienced two complications or more. Gender, income status, and number of complications were all associated with PTG scores, and female patients had higher PTG scores compared to male patients. Also, hemodialysis patients whose monthly income ≥10,000 Yuan had higher PTG than those with a monthly income of 5,000–10,000 Yuan and <5,000 Yuan. Hemodialysis patients with one complications reported higher PTG scores than other groups.

### Correlations among forgiveness, social support, and post-traumatic growth

Correlations, means, and deviations of the related variables are shown in Table 2. The mean scores of the PTG scales are moderate based on the judgment criteria of Su et al. (11). The mean score of perceived social support was 4.63, denoting a moderate level of social support (17). The results of the correlation analysis show that the study variables, forgiveness, social support, and PTG, were significantly correlated with each other, but the correlations with PTG were the strongest (Table 2).

**TABLE 2:** The Pearson correlation among forgiveness, social support, and post-traumatic growth (PTG).

Variable	Mean	SD	1	2	3
1 Forgiveness	4.53	0.71	1		
2 Social support	4.63	1.37	0.309**	1	
3 PTG	3.87	0.79	0.330**	0.466**	1

**P < 0.01.

### Regression analysis of forgiveness, social support, and post-traumatic growth

The HMR models of PTG are shown in Table 3. The final model explained a total of 27.6% of the variance in PTG. Gender, income status, and number of complications were significantly associated with PTG in model 1. According to the *R*^2^ change, forgiveness contributed to the total variance of PTG (10.3%), and social support was responsible for 12.9% of total variance of PTG. Forgiveness and social support were the strong predictors of PTG.

**TABLE 3:** The hierarchical multiple regression (HMR) models of post-traumatic growth (PTG).

	Model 1		Model 2		Model 3	
			
	β	95% CI	β	95% CI	β	95% CI
**Block 1 demographic characteristics and disease-related Information**						
Gender (male vs. female)	0.111*	0.037∼0.320	0.108*	0.040∼0.307	0.091*	0.022∼0.269
**Income status**						
(<5,000 Yuan vs. 5,000–10,000 Yuan)	0.100*	0.025∼0.393	0.080	–0.008∼0.341	0.062	–0.033∼0.289
(<5,000 Yuan vs. ≥10,000 Yuan)	0.069	–0.085∼0.715	0.048	–0.164∼0.595	0.024	–0.243∼0.458
**Number of complications**						
(0 vs. 1)	0.137*	0.064∼0.495	0.144**	0.091∼0.499	0.126**	0.068∼0.445
(0 vs. ≥2)	–0.019	–0.199∼0.138	–0.010	–0.175∼0.144	0.007	–0.136∼0.159
Block 2 forgiveness			0.322**	0.265∼0.449	0.209**	0.142∼0.321
Block 3 social support					0.380**	0.174∼0.568
*R* ^2^	0.044		0.147		0.276	
Adjust *R*^2^	0.034		0.136		0.266	
Δ*R*^2^	0.044		0.103		0.129	

**P < 0.01, *P < 0.05.

### Mediator of social support between forgiveness and post-traumatic growth

**FIGURE 2:**
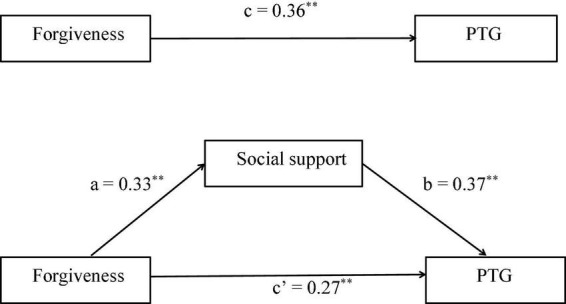
c: association of forgiveness with post-traumatic growth (PTG); a: association of forgiveness with social support; b: association between social support and PTG; c’: association of forgiveness with PTG after adding social support as a mediator. ^**^Indicating the coefficient of the path is significant.

The direct pathway between forgiveness and PTG is shown in Figure 2. The SEM model illustrated forgiveness had a significant direct influence on PTG (*c* = 0.36, *P* < 0.01). The SEM implied that forgiveness was positively associated with PTG and this model had a good model fit indices (RMSEA = 0.068, SRMR = 0.072, TFI = 0.959, CFI = 0.967, GFI = 0.952, and *x*^2^/d*f* < 5).

The mediating role of social support in the association between forgiveness and PTG is shown in Figure 2. Social support was significantly and positively associated with PTG (β = 0.37, *P* < 0.01). Moreover, when social support was modeled as a mediator, the path coefficient of forgiveness on PTG decreased significantly (*c*’ = 0.27, *P* < 0.01). The bias-corrected and accelerated bootstrap test showed that social support significantly mediated the relationship between forgiveness and PTG (a*b = 0.122, BCa 95% CI: 0.078∼0.181), which verified a significant partial mediating role of social support in the association between forgiveness and PTG. The model presented in Figure 2 was fully backed up by all standard goodness of fit indices (RMSEA = 0.072, SRMR = 0.076, TFI = 0.932, CFI = 0.958, GFI = 0.948, and *x*^2^/d*f* < 5). Thus, forgiveness directly influenced PTG and also influenced PTG indirectly by the mediating pathway of social support.

## Discussion

The direct effect of forgiveness on PTG was confirmed, and initial evidence of a mediating role of social support in the effects of forgiveness on PTG was found. These findings reveal new ways to improve PTG in hemodialysis patients by emphasizing the combined roles of forgiveness and social support.

Forgiveness was positively associated with PTG in hemodialysis patients. In line with previous findings (11, 12), our results show the helpful role that forgiveness could play in PTG. From a psychosocial perspective, forgiveness may serve as an adaptive emotion-focused coping strategy that has a beneficial role in managing negative emotions such as anger, anxiety, and depression (38). Some studies show that the more forgiving individuals are, the more selfregulated and able to manage challenges they become (39, 40). In addition, such individuals are more inclined to inhibit behavior that decreases their quality of life and are more inclined to abandon intrapersonal and/or interpersonal strategies such as hitting, using physical violence, and berating. Novak et al. (39) reported that trait forgiveness is regarded as a primary aspect of motivation transformation, which is viewed as operational in suppressing negative instincts and enhancing positive actions. Forgiveness has been closely tied to increased parasympathetic tone, decreased sympathetic arousal, improved physical health, and longevity and thus is viewed as an important psychological means to manage strong negative emotions and restore hope (41–43). Hemodialysis patients experience self-perceived economic, emotional, and physical burdens (44, 45). Low levels of forgiveness are linked to severe symptoms of anger, anxiety, depression, and post-traumatic stress disorder (38).

Social support had a positive and significant association with PTG, which is consistent with the results of earlier research (46, 47). For instance, Baillie et al. (48) found a higher level of social support to be associated with greater PTG. Social support is a multidimensional concept that refers to the provision of emotional, informational, and tangible assistance from others and involves respect, love, and acceptance for an individual (49). Social support may provide a platform for individuals to discuss and share their feelings, seek advice and make sense of the essence and significance of traumatic events, and ultimately positively reconstruct their assumptions (50, 51). A higher level of social support can increase individuals’ tolerance of distress when dealing with traumatic events (11). A socially supportive environment can provide not only a safe environment for individuals but also the necessary resources such as information and financial aid for individual responses, encouraging individuals to think positively about traumatic events and triggering an individual integration of the new meaning of traumatic events (52). Therefore, social support and a supportive social atmosphere could facilitate the successful management of difficulties and cognitive adaptation processes during traumatic experiences (46, 53), and this process could lead to the development of PTG.

This study not only verifies the direct relationship between forgiveness and PTG but is also the first to confirm the mediating effect of social support on the relationship between forgiveness and PTG. Social support strengthened the positive effect of forgiveness on PTG. In other words, the relationship between forgiveness and PTG was enhanced due to social support. Individuals who are more forgiving experience more PTG when they encounter traumatizing events and can actively seek positive resources such as social support (29). A mixed model of PTG shows that social support plays a positive role in PTG development (54). Forgiveness can motivate individuals to repeatedly think about traumatic events and change their cognition. This process is good for reframing beliefs about oneself, others and situations, thus helping individuals seek and perceive more social support (55). In addition, social support may not only encourage the expression of concerns but also provide the necessary information for discussion and determination, and this process facilitates growth (56). Therefore, forgiveness could cause individuals to perceive more social support, which could help them identify the positive aspects of traumatic events and redefine their post-traumatic experiences, thereby facilitating the achievement of PTG (57).

Among demographic characteristics and disease-related information, gender, income status and the number of complications were significant predictors of PTG. Women experienced more PTG than men, which is consistent with the results of earlier research (50). Women tend to use emotion-focused coping styles, such as positive reappraisal, reflection and positive self-talk, which involve rethinking traumatic events and attempting to make sense of them (58), thereby facilitating PTG. Income status had a positive effect on PTG, which echoes the findings of a previous study that found that individuals with more income experienced more PTG, as low-income individuals can often only afford low-quality treatment, resulting in more stress from traumatic events (59). The number of complications had a negative effect on PTG, potentially becomes some symptoms, such as itching, fatigue, restless legs syndrome, and depression, could lead to a significant decline in psychological, social, and physical function (60, 61).

## Conclusion

Our results verify that forgiveness has a direct effect on PTG and highlight the mediating effects of social support on the relationship between forgiveness and PTG, which has never been fully explored in hemodialysis patients. The practical implications of this study are obvious, especially for Chinese nurses. The findings indicate that forgiveness is a critical problem for hemodialysis patients. The forgiveness tendencies of hemodialysis patients should be assessed and monitored appropriately. Available psychological counseling services should be provided to hemodialysis patients when they experience severe symptoms of anxiety, depression, and post-traumatic stress disorder. As a preventive measure, interventions focused on ameliorating forgiveness with an emphasis on enhancing social support strategies could be developed and provided to hemodialysis patients.

## Limitation

Several limitations should be considered in interpreting our findings. First, the cross-sectional study design and questionnaire of self-reported nature prevented us from drawing directional conclusions about the relationships between forgiveness, social support, PTG, demographic characteristics, and disease-related information. Second, our convenience sampling (from nine hospitals) might limit the generalizability of our findings. Longitudinal research is crucial to replicate this study at different hospitals to confirm our results.

## Data availability statement

The original contributions presented in this study are included in the article/supplementary material, further inquiries can be directed to the corresponding author.

## Ethics statement

The Committee of Medical Ethics of the Sixth Affiliated Hospital of Kunming Medical University approved this research (Ethics number: 2020 kmykdx6937). The participants provided their written informed consent to participate in the study.

## Author contributions

YY was involved in the design of this study, data collection, analysis and interpretation of data, drafting, and revising the manuscript. ZT, CL, XG, YS, JX, QX, and CS provided help with the data collection, analysis, and interpretation. All authors had read and approved the final manuscript.
